# Knowledge of Oral Emergency Contraception Among Pharmacy Students

**DOI:** 10.1089/whr.2023.0175

**Published:** 2024-01-23

**Authors:** Bria Nikole Blake, Samantha Bookbinder, Gweneth Lazenby, Amari Marshall, Elizabeth Weed, Michelle Meglin

**Affiliations:** ^1^ Department of Obstetrics and Gynecology, Medical University of South Carolina, Charleston, South Carolina, USA.; ^2^ College of Medicine, Medical University of South Carolina, Charleston, South Carolina, USA.; ^3^ College of Pharmacy, Medical University of South Carolina, Charleston, South Carolina, USA.; ^4^ Department of Clinical Pharmacy and Outcome Sciences, Medical University of South Carolina, Charleston, South Carolina, USA.

**Keywords:** emergency contraception, pharmacy students, levonorgestrel, ulipristal acetate, pharmacy education

## Abstract

**Background:**

Access to emergency contraception is an important consideration in preventing unintended pregnancies. Inconsistent information about emergency contraceptive given to patients at retail pharmacies may limit access.

**Objective:**

In this study, we aimed to assess pharmacy students' knowledge of oral emergency contraception.

**Methods:**

Students in a Doctor of Pharmacy program completed a confidential survey about their knowledge of and training on oral emergency contraception. Respondents self-reported demographics included age, race, ethnicity, gender, and year in pharmacy school. The survey questions assessed student knowledge of indications, availability, side effects, and mechanisms of action of oral emergency contraception, as well as their training on emergency contraception. Chi-squared and Fisher's exact tests were used to determine if demographics influenced knowledge outcomes. A multivariate logistic regression, including age, gender, ethnicity, religion, year of training, hours of education, and source of knowledge acquisition, was used to adjust for confounding variables.

**Results:**

Among 296 pharmacy students, 31% (92/296) completed the survey. Among respondents, 34% (31/92) showed adequate knowledge of oral emergency contraception based on four critical knowledge questions. Third- and fourth-year students were more likely to have adequate knowledge than first- and second-year students (odds ratio [OR], 2.70; confidence interval [95% CI], 1.07–6.80). Students who reported learning about emergency contraception through reading assignments were more likely to have adequate knowledge than students who did not report learning from reading assignments (OR, 2.09; 95% CI, 1.30–3.35).

**Conclusions:**

Most pharmacy students at a single academic center did not have adequate knowledge of oral emergency contraception. These findings highlight the need for trainings to improve pharmacy student knowledge of oral emergency contraception.

## Introduction

In the United States, nearly half of pregnancies are unintended. However, between 2008 and 2011, the rate of unintended pregnancy declined from 51% to 45%, which may be explained by a greater use of contraception during this time.^1^ Interestingly, many unintended pregnancies occur even when patients are using contraception. Among more than 10,000 patients who obtained abortions, 54% were using contraception during the month they became pregnant.^2^

When contraception is not used or fails, emergency contraception can be used after vaginal intercourse to potentially prevent pregnancy. As a preventive measure, emergency contraception does not negatively impact an existing pregnancy and does not cause miscarriage or abortion.^3,4^ Emergency contraception was originally available only with a prescription, but the treatment is now also available over the counter. This change has been associated with greater patient access to emergency contraception. Indeed, among women 15 to 44 years of age, who have been sexually active, lifetime use of emergency contraception rose from 11% in 2008 to 23% in 2015.^5^ Thus, access to emergency contraception is an important consideration in preventing unintended pregnancies.

Oral emergency contraception is safe and effective with very few contraindications. The US Federal Drug Administration has approved several forms of emergency contraception, which can potentially prevent pregnancy, including oral levonorgestrel and oral ulipristal acetate. Oral levonorgestrel 1.5 mg single dose (Plan B One Step) is labeled for use within 72 hours after vaginal intercourse and is available without a prescription or age restriction. Oral ulipristal acetate single dose (Ella) can be taken up to 5 days after intercourse, is available only by prescription, and is available without an age restriction. Both of these medications work by delaying ovulation.^3,4^

When purchasing oral emergency contraception, patients often only encounter pharmacists and no other medical professional. However, pharmacists and pharmacy technicians in retail pharmacies provide inaccurate information about purchasing or using oral emergency contraception >50% of the time.^6^ This misinformation may be due to pharmacists' inadequate knowledge of emergency contraception.^7^ In this study, we aimed to assess pharmacy students' knowledge of and training on oral emergency contraception. We hypothesized that pharmacy students do not have adequate knowledge of and training on emergency contraception, particularly ulipristal acetate. We also posited that most pharmacy students are unaware of unrestricted access (no age or gender criteria) to purchase oral levonorgestrel single dose.

## Methods

This cross-sectional study included pharmacy students across all 4 years of pharmacy school in an academic medical center located in the southeastern United States. All students enrolled in the Doctor of Pharmacy program were invited to complete a one-time, anonymous survey about their knowledge of and training in oral emergency contraception. Students received a link to the REDCap survey through their school email address on three occasions over a 3-month period (November 2021 to January 2022). Students were assured that their responses were anonymous, not linked to their email addresses, and would not affect their personal or academic standing. Students were also told that the survey results could improve curriculum development at their institution. Consent was obtained at the beginning of the survey.

Respondents self-reported demographic characteristics, including age, race, ethnicity, gender identity, year in pharmacy school, religious affiliation, number of hours of formal education on emergency contraception, and what sources they used to learn about emergency contraception. These demographic characteristics were collected because age,^8^ gender,^9–11^ ethnicity,^9^ religion,^9,11–13^ year of training,^10,14^ hours of education,^14^ and source of knowledge acquisition^14^ have been associated with differences in student, resident, and pharmacist knowledge and/or pharmacist practice patterns. Pharmacy student knowledge of the availability, side effects, mechanisms of action, and purchasing access was assessed through 24 questions (20 true/false and 4 multiple choice). The questions were adapted from prior studies of (1) emergency contraception knowledge and training among residents in obstetrics/gynecology and family medicine, and (2) emergency contraception knowledge among adolescents.^8,14^

Adequate knowledge of emergency contraception was defined as correctly answering four key knowledge questions: (1) “Oral forms of emergency contraception work by causing a miscarriage or abortion.” (true/false); (2) “For how long after intercourse is levonorgestrel (Plan B One Step) FDA approved?” (24/48/72/96/120 hours); (3) “How long after intercourse is ulipristal acetate (Ella) considered to be effective?” (24/48/72/96/120 hours); and (4) “A woman must take a pregnancy test before taking levonorgestrel (Plan B One Step) or ulipristal acetate (Ella).” (true/false). These questions were deemed most likely to impact a patient's ability to obtain oral emergency contraception from a retail pharmacy.

Due to our small sample size, the following variables were dichotomized: age, race, ethnicity, year of training, religion, and hours of education. Chi-squared and Fisher's exact tests were used to evaluate associations between demographic variables and knowledge outcomes. Univariate logistic regression was used on significant variables to obtain odds ratios. Age, gender, ethnicity, religion, year of training, hours of education, and source of knowledge acquisition were included as covariates in a multivariate logistic regression model to generate adjusted odds ratios. Data analysis was performed with Stata version 16. This study was determined to be exempt from IRB review by the Medical University of South Carolina Institutional Review Board.

## Results

Among 296 enrolled pharmacy students, 92 (31%) completed the survey. Among respondents, 45% were in their first or second year of pharmacy school, and 55% were in their third or fourth year of pharmacy school. Most respondents self-identified as White (83%), and a small percentage self-identified as Asian (4%), Black (15%), Hispanic (4%), or belonging to other racial or ethnic groups (2%). No respondent identified as American Indian or Alaskan Native. Also, most respondents self-identified as cisgender women (87%). The remainder self-identified as cis-gender men (12%) and nonbinary (1%). No respondent identified as transgender man or transgender woman. Most respondents were 20 to 25 years old (74%) and self-identified as Christian (71%) (Table 1).

**Table 1.** Respondent Characteristics by Adequacy of Knowledge (*N* = 92)

Demographic	Adequate knowledge, ***n*** (%) (***n*** = 31)	Inadequate knowledge, ***n*** (%) (***n*** = 61)	***p***
Age			0.64
≤ 25 years old	22 (71)	46 (75)	
> 25 years old	9 (29)	15 (35)	
Race			0.25
Non-White	4 (13)	14 (23)	
White	27 (87)	47 (77)	
Ethnicity			0.70
Hispanic	1 (3)	3 (5)	
Non-Hispanic	30 (93)	58 (95)	
Gender			0.28
Man	6 (19)	5 (8)	
Woman	25 (81)	55 (90)	
Nonbinary	0 (0)	1 (2)	
Transgender man or woman	0 (0)	0 (0)	
Years of training			0.03
1 or 2	9 (29)	32 (52)	
3 or 4	22 (71)	29 (48)	
Religion			0.31
Christian	24 (77)	41 (67)	
Non-Christian	7 (23)	20 (33)	
Education			0.49
≤1 hour	17 (55)	38 (62)	
≥2 hours	14 (45)	23 (38)	
Learned about EC from lectures			0.19
Yes	27 (87)	46 (75)	
No	4 (13)	15 (25)	
Learned about EC from assigned reading			0.002
Yes	16 (52)	12 (20)	
No	15 (48)	49 (80)	
Learned about EC from another source			0.60
Yes	11 (35)	25 (41)	
No	20 (65)	36 (59)	

EC, emergency contraception.

Among respondents, 34% had adequate knowledge of oral emergency contraception, defined as correctly answering four critical knowledge questions (Table 2). Third- and fourth-year students were more likely to have adequate knowledge than first- and second-year students (odds ratio [OR], 2.70; confidence interval [95% CI], 1.07–6.80). Among third- and fourth-year pharmacy students, 43% had adequate knowledge of emergency contraception. Students who reported learning about emergency contraception through reading assignments were more likely to have adequate knowledge than students who did not report learning from reading assignments (OR, 2.09; 95% CI, 1.30–3.35). In the adjusted model, adequate knowledge was only associated with learning from reading assignments (adjusted OR [aOR], 1.99; 95% CI, 1.23–3.22). Adequate knowledge was not associated with self-reported age, race, ethnicity, gender, religion, number of hours of education, learning about emergency contraception from lecture, or learning from another source.

**Table 2.** Responses to Critical Knowledge Questions (*N* = 92)

Oral forms of emergency contraception work by causing a miscarriage or abortion.				
True	False^a^			
3 (3)	89 (97)			
For how long after intercourse is levonorgestrel (Plan B One Step) FDA approved?				
24 hours	48 hours	72 hours^a^	96 hours	120 hours
2 (2)	8 (9)	76 (83)	1 (1)	5 (5)
How long after intercourse is ulipristal acetate (Ella) considered to be effective?				
24 hours	48 hours	72 hours	96 hours	120 hours^a^
7 (8)	16 (17)	20 (22)	12 (13)	37 (40)
A woman must take a pregnancy test before taking levonorgestrel (Plan B One Step) or ulipristal acetate (Ella).				
True	False^a^			
2 (2)	90 (98)			

Questions adapted from Rapkin et al.^14^ and presented as *n* (%).

^a^ Correct answer.

FDA, Federal Drug Administration.

Approximately one-quarter of pharmacy students correctly identified the primary mechanism of action of levonorgestrel (21%) and ulipristal acetate (25%) (Table 3). Their correct answers to 11 additional safety and side effect questions ranged from 32% to 97% (Table 3). Most pharmacy students correctly answered questions about access to oral levonorgestrel (77% to 98%), but their answers were more varied for questions about access to ulipristal acetate (41% to 78% correct) (Table 4).

**Table 3.** Responses to Pharmacology Questions (*N* = 92)

Which of the following is the primary mechanism of levonorgestrel (Plan B One Step)?				
Altering tubal transport of sperm and/or ova to prevent fertilization 10 (11)	Altering the endometrium to prevent implantation 57 (62)	Delaying ovulation^a^ 20 (22)	Causing embryos to be expelled after implantation 2 (2)	Creating a sterile inflammatory reaction that is toxic to sperm and ova 3 (3)
Which of the following is the primary mechanism of ulipristal acetate (Ella)?				
Altering tubal transport of sperm and/or ova to prevent fertilization 17 (19)	Altering the endometrium to prevent implantation 35 (38)	Delaying ovulation^a^ 23 (25)	Causing embryos to be expelled after implantation 10 (11)	Creating a sterile inflammatory reaction that is toxic to sperm and ova 7 (8)
Levonorgestrel (Plan B One Step) and ulipristal acetate (Ella) are safe to prescribe to women who have hypertension			True^a^ 66 (72)	False 26 (28)
Levonorgestrel (Plan B One Step) and ulipristal acetate (Ella) are safe to prescribe to women who are breastfeeding			True^a^ 29 (32)	False 63 (68)
Levonorgestrel (Plan B One Step) and ulipristal acetate (Ella) are safe to prescribe to women who are smokers			True^a^ 50 (54)	False 42 (46)
Common side effects of levonorgestrel (Plan B One Step) and ulipristal acetate (Ella) are disruption of the menstrual cycle			True^a^ 89 (97)	False 3 (3)
Common side effects of levonorgestrel (Plan B One Step) and ulipristal acetate (Ella) are headache			True^a^ 84 (91)	False 8 (9)
Common side effects of levonorgestrel (Plan B One Step) and ulipristal acetate (Ella) are infertility			True 11 (12)	False^a^ 81 (88)
Common side effects of levonorgestrel (Plan B One Step) and ulipristal acetate (Ella) are nausea			True^a^ 90 (98)	False 2 (2)
Levonorgestrel (Plan B One Step) and ulipristal acetate (Ella) can increase a woman's risk of DVT			True 55 (60)	False^a^ 37 (40)
Levonorgestrel (Plan B One Step) and ulipristal acetate (Ella) can cause birth defects if taken while a woman is pregnant			True 52 (57)	False^a^ 40 (43)
Levonorgestrel (Plan B One Step) and ulipristal acetate (Ella) can only be used once per menstrual cycle			True 21 (23)	False^a^ 71 (77)
Levonorgestrel (Plan B One Step) and ulipristal acetate (Ella) can make it difficult to have pregnancies in the future			True 10 (11)	False^a^ 82 (89)

Questions adapted from Rapkin et al.^14^ and presented as *n* (%).

^a^ Correct answer.

**Table 4.** Responses to Access Questions (*N* = 92)

Levonorgestrel (Plan B One Step) can only be sold to women older than 18 years of age	True 20 (22)	False^a^ 70 (78)
Levonorgestrel (Plan B One Step) can only be purchased with a prescription from a health care provider	True 2 (2)	False^a^ 90 (98)
Levonorgestrel (Plan B One Step) can be sold to anyone (male or female) regardless of age	True^a^ 71 (77)	False 21 (23)
Ulipristal acetate (Ella) can only be prescribed to women older than 18 years of age	True 46 (50)	False^a^ 46 (50)
Ulipristal acetate (Ella) can only be prescribed to women younger than 18 years of age with parent permission	True 54 (59)	False^a^ 38 (41)
Ulipristal acetate (Ella) can only be purchased with a prescription from a health care provider	True^a^ 72 (78)	False 20 (22)

Questions adapted from Williams et al.^8^ and presented as *n* (%).

^a^ Correct answer.

## Discussion

In this study, we assessed pharmacy students' knowledge of and training on oral emergency contraception at a single academic center. We found that most pharmacy students did not have adequate knowledge of oral emergency contraception and could not correctly identify the recommended timing for administering oral ulipristal acetate after intercourse. Only one-quarter of pharmacy students correctly identified the primary mechanism of action of oral emergency contraception.

The majority of students (62%) erroneously identified alteration of the endometrium to prevent implantation as the primary mechanism of action of oral levonorgestrel. In addition, most students incorrectly thought oral emergency contraception was not safe for breastfeeding women (68%) and increased a woman's risk of venous thromboembolism (60%). In contrast, most students had knowledge of unrestricted access (no age or gender criteria) to purchasing oral levonorgestrel single dose. Also, students were more likely to have adequate knowledge when they reported learning from required reading versus lectures. These findings suggest that pharmacy students at our institution did not have adequate knowledge of and training in oral emergency contraception.

Several cross-sectional studies found inconsistent knowledge and beliefs about emergency contraception among practicing pharmacists. Some studies found that pharmacists had low knowledge of oral emergency contraception,^7,12^ and others showed average^9^ and high knowledge of emergency contraception.^13,15^ This inconsistency may be due to the different questions that these studies used to assess knowledge. For example, Griggs and Brown^13^ found that pharmacists in Texas were quite knowledgeable. However, this knowledge was defined as having heard of oral emergency contraception and knowing oral levonorgestrel was most effective if taken within 72 hours.

On the other hand, El-Ibiary et al.^15^ found that retail pharmacists in San Francisco had high knowledge of treatment protocols and emergency contraception access. However, their 11-question assessment was done after an optional training on emergency contraception, which may have contributed to the higher knowledge. Our study evaluated several domains of knowledge, including the timing of oral emergency contraception after intercourse, the lack of requirement for a pregnancy test, and that emergency contraception does not cause an abortion.

Several researchers have raised concerns about pharmacy students' knowledge of emergency contraception.^10,16^ Young et al.^11^ found that pharmacy students at the University of Arkansas had several knowledge deficits related to oral levonorgestrel. Like our findings, only one-third of students knew the mechanism of action of oral levonorgestrel. Young et al. also found that 35% of pharmacy students incorrectly identified oral levonorgestrel as mifepristone (RU486), an oral medication used for medication abortion. In contrast, 97% of students in our study recognized that oral emergency contraception does not cause abortion. In another study, Evans et al.^10^ found gaps in pharmacy students' knowledge of emergency contraception. They also found that pharmacy students with more knowledge were more likely to support emergency contraception and had fewer concerns with its use.

This is one of the first studies to evaluate knowledge of ulipristal acetate and levonorgestrel emergency contraception rather than levonorgestrel only. In our study, only 40% of pharmacy students correctly answered timing of ulipristal acetate use compared to 83% for timing of levonorgestrel use. In addition, pharmacy students were less likely to correctly answer questions about access to ulipristal acetate compared to access to levonorgestrel.

Knowledge of emergency contraception may also affect access to emergency contraception. Indeed, one study found that greater knowledge of emergency contraception was the most important predictor of pharmacists' dispensing emergency contraception.^17^ This finding implies that enhancing knowledge of emergency contraception may improve patient access to this treatment option. However, more research is needed to determine the interventions that are most likely to improve knowledge of emergency contraception and how these improvements may impact future practice.

One way to improve pharmacy students' knowledge of emergency contraception is to create training opportunities for these students. For example, after a one-time counseling workshop, pharmacy students improved their knowledge of emergency contraception (from 86% to 93%) and confidence in counseling patients on emergency contraception (from 26% to 59%). Also, the percentage of students who believed that nonprescription emergency contraception would promote unsafe sex decreased after the workshop (21% to 14%).^15^ At the time of our survey, first- and second-year pharmacy students at our institution received a total of two lecture hours on all types of contraception, including emergency contraception. To enhance our training program, we propose to increase didactic instruction using a flipped classroom model and interactive counseling sessions with standardized patients.^15,18^

Our study has several notable strengths. First, our survey included validated questions that were left unchanged or only required minor revisions. Also, the survey assessed pharmacology, access, and both types of oral emergency contraception (levonorgestrel and ulipristal acetate). Finally, our study comprised a diverse multidisciplinary team of medical and pharmacy students, as well as practicing obstetrician gynecologists and a Doctor of Pharmacy.

Our study also has a few important limitations. For example, we had a small sample size due to a low response rate to our survey. Due to the possibility of nonresponse bias, our findings may not be representative of the entire pharmacy student population. In addition, our sample included small numbers of respondents who identified as certain racial, ethnic, and religious groups, which required creating dichotomous categories in the analysis. Evaluation of student knowledge was limited to the specific questions asked. Unfortunately, we did not assess knowledge of risk factors for oral emergency contraceptive failure such as body mass index greater than 25 kg/m^2^.^19^ Finally, our study lacked an educational intervention. However, our findings can serve as a baseline to inform future efforts for enhancing pharmacy student training on emergency contraception.

## Conclusion

In conclusion, we found that most pharmacy student respondents at our institution did not have adequate knowledge of oral emergency contraception. Adequate knowledge was associated with more years of education and knowledge obtained from reading, but not with demographic characteristics, such as race, ethnicity, gender, and religion. These findings highlight that pharmacy schools need to enhance training programs for pharmacy students to improve their knowledge of emergency contraception. This improved knowledge will help pharmacists to better serve patients seeking emergency contraception to prevent unintended pregnancy.

## Abbreviations Used

aOR: adjusted odds ratio
CI: confidence interval
OR: odds ratio
